# Feasibility and acceptability of therapist-guided, asynchronous, internet-delivered trauma-focused CBT for adolescents with PTSD: a single-group feasibility trial in Sweden

**DOI:** 10.1136/bmjopen-2026-117024

**Published:** 2026-05-27

**Authors:** Erica Mattelin, Hanna Weyler, Rebecca Andersson, Josefine Paulsen, Sandra Tielman, Anna Vikgren, Kristina Bondjers, Eva Serlachius, David Mataix-Cols, Maria Bragesjö

**Affiliations:** 1 Centre for Psychiatry Research, Department of Clinical Neuroscience, Karolinska Institutet, and Stockholm Health Care Services, Region Stockholm, Sweden; 2 Save the Children, Stockholm, Stockholm County, Sweden; 3 Department of Clinical Neuroscience, Karolinska Institute Centre for Psychiatry Research, Stockholm, Sweden; 4 National Centre for Disaster Psychiatry, Uppsala University Department of Medical Sciences, Uppsala, Uppsala County, Sweden; 5 Department of Clinical Neuroscience, Karolinska Institute Centre for Psychiatry Research, Stockholm, Stockholm County, Sweden; 6 Department of Clinical Sciences, Lund University Faculty of Medicine, Lund, Skåne County, Sweden; 7 Department of Child and Adolescent Psychiatry, Skåne University Hospital Lund, Lund, Skåne County, Sweden; 8 Department of Child and Adolescent Psychiatry, Aarhus University Hospital Psychiatry, Aarhus, Central Denmark Region, Denmark

**Keywords:** Stress Disorders, Traumatic, Acute, Adolescents, Child & adolescent psychiatry, Gender-Based Violence, Internet, Digital Technology

## Abstract

**Objectives:**

Trauma-focused cognitive behavioural therapy (TF-CBT) is the established first-line treatment for paediatric post-traumatic stress disorder (PTSD), but access to evidence-based care remains limited. This study aimed to evaluate the feasibility and acceptability of a therapist-guided, 12 week, internet-delivered TF-CBT (iTF-CBT) programme for adolescents with PTSD and to explore preliminary changes in PTSD symptoms.

**Design:**

Single-group feasibility trial.

**Setting:**

Save the Children, Sweden.

**Participants:**

Twenty-two adolescents (13–17 years, 82% female) with primary PTSD.

**Interventions:**

A 12 week, therapist-guided, asynchronous, internet-delivered TF-CBT comprising eight modules and parallel caregiver modules with joint adolescent–caregiver activities.

**Outcomes:**

Feasibility measures included recruitment pace, participant retention, treatment adherence (module completion) and therapist time. Acceptability was evaluated through satisfaction, credibility, negative effects and reported adverse events. Within-group changes in PTSD severity using independent evaluator-rated Clinician-Administered PTSD Scale (CAPS-CA-5) and the self-reported Child and Adolescent Trauma Screen 2 (CATS-2) were used as indicators of potential clinical change. Assessments occurred at baseline, during treatment, post-treatment and at 1 month follow-up (primary endpoint).

**Results:**

Recruitment was completed after 7 months of active enrolment. Retention and adherence were high, satisfaction and credibility ratings were favourable, and no intervention-related serious adverse events occurred. Within-group improvements were observed at the primary endpoint, with large reductions on CAPS-CA-5 (Cohen’s d=1.27) and CATS-2 (Cohen’s d=1.51). At follow-up, 47.6% of participants no longer met criteria for PTSD.

**Conclusions:**

Therapist-guided iTF-CBT for adolescents with PTSD was safe, feasible, acceptable and associated with potentially meaningful symptom improvements. These findings support further evaluation in larger, controlled trials to determine efficacy, cost-effectiveness and long-term outcomes.

**Trial registration number:**

NCT06185244.

**STRENGTHS AND LIMITATIONS OF THIS STUDY**

- Single-group feasibility trial evaluating recruitment, adherence, acceptability and safety of internet-delivered trauma-focused cognitive behavioural therapy for adolescents with post-traumatic stress disorder (PTSD).
- Use of independent evaluators and the clinician-administered Clinician-Administered PTSD Scale (CAPS-CA-5) interview to assess PTSD severity.
- Delivery within a routine specialist clinic setting with nationwide recruitment.
- Lack of a control group limits conclusions regarding treatment effectiveness.

## Introduction

 Potentially traumatic events are common in young people, with studies indicating that a substantial proportion have experienced at least one such event by late adolescence.^1 2^ Among those exposed, the prevalence of post-traumatic stress disorder (PTSD) is estimated at 5%–16%.^3^

PTSD is a debilitating condition characterised by intrusive memories, avoidance of trauma reminders, negative changes in cognition and mood, and hyperarousal, and is associated with marked functional impairment and psychiatric comorbidity.^4^ Untreated PTSD in youth substantially increases the risk of substance use, suicidality, poor mental health and academic underachievement.^5^,^7^

Trauma-focused cognitive behavioural therapy (TF-CBT) is a first-line treatment for paediatric PTSD,^8^ yet many adolescents do not receive treatment because of structural (limited clinician availability, waiting lists), logistical (travel, scheduling) and psychological (stigma, avoidance, caregiver burden) barriers. One possible solution to the limited availability of specialised TF-CBT for youth with PTSD is to deliver a low-intensity version of the treatment online with minimal remote support from a clinician. This kind of guided internet-delivered CBT differs from standard telepsychiatry in that the therapist does not actively deliver the treatment content in real time. Instead, the therapist provides minimal support asynchronously via a messaging system built in the online platform.

Adult trials suggest that therapist-guided internet-delivered CBT can achieve meaningful PTSD reductions and, in some settings, outcomes comparable to face-to-face care.^9^,^11^ Similarly, internet-delivered CBT has shown efficacy across several child and adolescent mental health conditions, including both asynchronous and synchronous therapist-guided interventions^12 13^ . However, therapist-guided internet-delivered TF-CBT (henceforth iTF-CBT) for adolescents with diagnosed PTSD remains understudied.^14^ Key uncertainties include feasibility (recruitment, retention and adherence), acceptability to adolescents and caregivers, therapist time and resource requirements, safety and comparative evidence on efficacy and cost-effectiveness based on clinician- and self-reported outcomes.

The primary aim of this study was to evaluate the feasibility and acceptability of a therapist-guided, 12 week iTF-CBT programme for adolescents with PTSD. The main outcomes were recruitment pace, participant retention, treatment adherence, user satisfaction and safety. Within-group analyses of clinical outcomes were used to provide an initial indication of potential clinical change and to assess whether the intervention appears suitable for further evaluation in a future randomised controlled trial (RCT).

## Methods

### Study design and setting

This single-site, single-group feasibility trial was delivered at a non-for-profit specialist clinic for children and young people (Save the Children Sweden). The clinic specialises in the assessment and treatment of vulnerable children exposed to violence, primarily presenting with child psychiatric problems such as PTSD. Referrals come from social services, schools and civil society organisations as well as through self-referral. Common to all cases is that the children have not accessed adequate care within the regular healthcare system.

### Participants

Inclusion criteria were 13–17 years of age; primary diagnosis of PTSD; both adolescents and caregivers fluent in Swedish; access to the Internet at home or ability to use study-provided prepaid mobile data vouchers; caregiver willing and able to take part in treatment. Exclusion criteria were psychosis or another primary psychiatric disorder of such severity requiring urgent specialist care or precluding participation (eg, acute mania, an eating disorder requiring medical monitoring); ongoing substance abuse; high suicide risk; initiation or adjustment of any psychotropic medication within the last four weeks prior to treatment start and ongoing psychological treatment for PTSD.

### Sample size

This pilot was designed to evaluate feasibility and acceptability, not to establish efficacy. Consistent with guidance for feasibility studies, no formal power calculation was undertaken. A pragmatic target of N = 22 was chosen to enable estimation of key feasibility parameters and safety monitoring. Clinical outcomes were prespecified as exploratory to provide preliminary effect size and variance estimates for a randomised trial.

### Primary outcome measures

#### Feasibility

Feasibility was assessed across four domains: study uptake, timeliness, data completeness and resource use/cost. Study uptake comprised the recruitment rate (participants included per active week), screening-to-enrolment yield and the proportion of eligible families who consented and initiated iTF-CBT and referral source (self-referred vs health care-signposted). Timeliness included the time between registration and the start of treatment. Data completeness was summarised at the 1 month primary endpoint. Resource use/cost quantified therapist workload, platform-logged outgoing messaging time per module and manually logged phone time and recruitment spend per enrolled participant.

#### Acceptability and satisfaction

Acceptability was indexed by module completion for adolescents and caregivers. Treatment discontinuation was defined a priori as completing fewer than four of 12 modules; conversely, completing ≥4/12 modules was taken to indicate receipt of the intervention’s core components. Acceptability was further measured by treatment satisfaction in both adolescents and caregivers using the Client Satisfaction Questionnaire (CSQ) at post-treatment.^15^ The CSQ-8 consists of eight items, yielding a total score ranging from 8 to 32, with higher scores indicating greater satisfaction. The Credibility/Expectancy Questionnaire (CEQ)^16^ was administered after the completion of treatment module 1. The questionnaire consists of six questions and ranges between 6 and 54, with higher scores indicating greater credibility.

#### Safety

Negative effects were measured with the Negative Effects Questionnaire (NEQ) by both adolescents and caregivers. NEQ^17^ consists of 20 items with a total range of 0–80, with higher values representing more reported negative events. In addition to NEQ, therapists were encouraged to report other adverse events (AEs) both at the weekly multidisciplinary team meetings but also directly to the research leader if they should occur during treatment. Safety was also monitored via a weekly platform-based suicidality item (‘I have thoughts about death and about killing myself’) with predefined escalation (same-day clinician review, risk assessment and referral). AEs and serious adverse events (SAEs) were recorded in the electronic health records, including whether they were judged to be treatment-related. AEs were broadly defined as any negative or undesirable experience reported by the participant during the intervention period. SAEs were defined as suicide attempts, serious violent incidents or admission to hospital in line with the study protocol.

### Secondary outcome measures

#### Clinician-rated outcomes

Changes in post-traumatic stress symptoms (primary measure of clinical efficacy) were assessed using the Clinician-Administered PTSD Scale for Children and Adolescents—Version 5 (CAPS-CA-5), a gold-standard clinical interview for diagnosing PTSD and evaluating symptom severity. Assessments were conducted at baseline and at 1 month follow-up. CAPS-CA-5 covers the 20 DSM-5 PTSD symptoms, associated distress/impairment, change since the prior assessment and the dissociative specifier. Higher scores denote higher severity and impairment.^18^

#### Self-report outcomes

All self-report secondary measures were administered via the digital platform. PTSD symptoms were assessed with the Child and Adolescent Trauma Screen—Version 2^19^ (CATS-2; range 0–60, higher scores representing greater symptom burden) at baseline, weekly during treatment, post-treatment and at 1 month follow-ups. Depressive symptoms were assessed using the Mood and Feelings Questionnaire^20^ (MFQ; 0–26, with higher scores indicating greater severity) at baseline, post-treatment and at 1 month follow-ups. Functional impairment was evaluated with both youth- and caregiver-reported versions of the Work and Social Adjustment Scale^21^ (WSAS-Y and WSAS-P; 0–40, higher scores reflecting greater impairment) across the same time points. Health-related quality of life was measured with the KIDSCREEN-10 Index (10–50, higher scores denoting better quality of life).^22^

### Recruitment and procedures

Potential participants self-referred via social-media adverts linking to study information and a secure, password-protected study portal. Study staff then called registrants to provide further information, obtain consent to screening and schedule an interview with a licensed psychologist (HW). Initial eligibility was checked against inclusion and exclusion criteria; non-eligible applicants were notified by phone and where appropriate referred to alternative health care services. Demographic information on adolescents (eg, age, trauma type, gender, current and past psychotropic medication and prior psychological treatment) was also collected while caregiver data were obtained separately through an online questionnaire. Eligible dyads completed baseline questionnaires online including CATS-2, MFQ, WSAS-Y/P, TIC-P, KIDSCREEN-10 prior to clinical assessment. Diagnostic assessment comprised the Mini International Neuropsychiatric Interview for Children and Adolescents (MINI-KID)^23^ to assess comorbidities and evaluate suicidality, and the CAPS-CA-5 to confirm PTSD and rate past-month severity. At this assessment, the patient’s own account of the traumatic event was documented and evaluated against DSM-5 trauma criteria (criterion A). The categorisation of the traumatic event was based on the patient’s self-reported description and was not independently coded (table 1). Interviews were conducted in person or via video/telephone by a clinical psychologist or trained psychology student. Baseline assessments additionally included suicide risk (MINI-KID) and global functioning with The Children’s Global Assessment Scale (CGAS).

**Table 1:** Baseline demographic and clinical characteristics of the sample

	Total
**Age, mean (SD), min–max**	16.06 (1.52), 13–17
Gender, n (%)	
Male	3 (13.6)
Female	18 (81.8)
Non-binary	1 (4.5)
**Main contact person, mothers, n (%**)	21 (95.5)
**Education of primary contact person n (%**)	
Secondary education	4 (13.6)
Postsecondary education (not tertiary/college)	3 (13.6)
Higher education (3 years or less)	4 (18.2)
Higher education (3<)	11 (50.0)
Current psychiatric comorbidity n (%)	
At least one psychiatric comorbidity*	22 (100)
ADHD/ASD	15 (68.2)
Type of trauma n (%)	
Sexual violence	5 (22.7)
Witnessed a medical emergency	2 (9.1)
Physical assault	4 (18.2)
Witnessed violence	3 (13.6)
Bullying	4 (18.2)
Other	4 (18.2)
**Number of reported traumatic events, mean (SD**)	4.36 (2.7)
**Current use of any psychotropic medication n (%**)	13 (59.1)
Suicidality,* n (%)	
Low risk	14 (63.6)
Medium risk	3 (13.6)
High risk	5 (22.7)

* Comorbidity and suicidality was assessed using the Mini International Neuropsychiatric Interview for Children and Adolescents (MINI-KID 7.0).

ADHD, attention-deficit/hyperactivity disorder; ASD, autism spectrum disorder.

Final inclusion was confirmed at weekly multidisciplinary case conferences. Treatment typically commenced the following week; some start dates were deferred over the summer to avoid interruption. Both clinician-rated and self-reported outcomes were assessed at baseline and 1 months assessment. Written informed consent (online supplemental material 1) was obtained from adolescents and their legal guardians. Non-eligible applicants were notified by phone and, if needed, referred to regular healthcare.

### Intervention

The iTF-CBT protocol mirrors first-line treatment for children with PTSD^24^ and is delivered via a secure digital platform over a 12-week programme. The programme comprises eight adolescent modules with parallel caregiver modules (table 2). Core components include psychoeducation, affect/emotion regulation, trauma narration and cognitive processing, in-vivo exposure, safety planning and relapse prevention. Treatment initially targets the most distressing trauma memory, with flexibility to address additional events as needed. To maintain continuity in graded sharing (ie, progressive disclosure of the trauma narration from outline to fuller detail as tolerated), we scheduled the joint caregiver–adolescent session prior to initiating in-vivo exposure.

**Table 2:** Overview of the treatment content*

Week	Adolescent	Caregiver	Joint
1	Psychoeducation Caregivers and adolescents learn about exposure to trauma and the potential effects	Caregiver skills and psychoeducation	Psychoeducation
2	Relaxation Relaxation techniques are taught to help manage stress and anxiety and to prepare for the trauma narration	Caregiver skills and relaxation	Relaxation
3	Affective modulation Involves identifying, labelling, expressing and coping with feelings.	Caregiver skills and affective modulation	Affective regulation
4	Cognitive coping Involves how thoughts, feelings and behaviours are connected and how we ourselves can influence these.	Caregiver skills and cognitive coping	Cognitive coping
5	Trauma narration and cognitive processing Revisiting trauma memory through narrative exposure and cognitive processing.	Caregiver skills and cognitive processing	Cognitive processing
6	Trauma narration and cognitive processing	Caregiver skills and cognitive processing	Cognitive processing
7	Trauma narration and cognitive processing	Caregiver skills and cognitive processing	Cognitive processing
8	Trauma narration and cognitive processing	Caregiver skills and cognitive processing	Cognitive processing
9	Joint adolescent–caregiver session Joint session in which the adolescent shares the whole trauma narrative	Caregiver skills and cognitive processing	Cognitive processing
10	In vivo Gradually confronting and overcoming fears and reminders of the trauma through real-life exposures	In vivo	In vivo
11	Enhancing safety and future development Safety planning and maintenance planning	Future safety	Future safety
12	Future safety	Future safety	Celebration

* All modules involve gradual exposure.

Modules use age-appropriate text, audio and brief video vignettes and are accessed sequentially (one module per week) with home practice tasks. Each adolescent and their caregiver/guardian were assigned a therapist who provided guidance primarily via asynchronous communication (ie, secure messaging) within the platform. Caregivers engage in parallel modules that mirror the adolescent’s content, including caregiving practices (such as praise and validation), with joint caregiver–adolescent activities integrated throughout. The intervention was delivered in Swedish.

### Therapists

Seven clinical psychologists/psychotherapists from Save the Children delivered the intervention. All had prior TF-CBT training (3–12 years of experience with the method) and received onboarding to the digital protocol and platform before treatment started. Therapists were expected to log in at least twice per week. The therapists attended weekly joint case conferences with the Principal Investigator (PI; MB) to secure treatment fidelity, monitor safety for the participants and identify adjustment needs.

### Assessors

Outcome assessments were conducted primarily by psychology students in the final semester of a 5 year master’s programme, all trained in CGAS, MINI-KID and CAPS-CA-5 prior to data collection. In addition, three therapists conducted a subset of assessments after completing the same training. Given the uncontrolled study design, outcome assessors were not blinded.

### Patient and public involvement

The study originated from clinical needs identified by practitioners. Clinicians and trainers in TF-CBT at Save the Children, drawing on more than a decade of experience providing trauma-focused support to young people exposed to violence, developed the intervention and its core components.

Young people, both with and without lived experience of PTSD, were involved in the development of the intervention through iterative feedback on content, language and usability. This included reviewing selected materials (eg, written content and videos) and participating in think-aloud sessions, in which they worked through parts of the intervention while providing real-time feedback. Although this involvement was limited in scope, it informed subsequent refinements to the intervention. Clinical perspectives were further incorporated through consultation with national clinical experts in trauma-focused treatment for children, clinical experts in iCBT and young people, who reviewed the intervention and provided feedback on its structure and clinical content. Throughout the project, child and adolescent psychiatry personnel and Save the Children clinicians contributed to identifying needs, prioritising treatment components and shaping the implementation of the study. Through this process, we made several refinements, including simplifying the text, adjusting the sequencing of components (eg, reordering sharing and in vivo exposure) and refining the tone, wording and examples.

While stakeholder input informed several stages of the intervention development, the feasibility study itself did not include a fully structured PPI process due to the absence of dedicated funding for such activities at this stage. More extensive patient and public involvement is planned for the subsequent RCT.

### Statistical methods

Feasibility and acceptability metrics were summarised descriptively. For the clinical outcomes, Cohen’s d statistics were derived from linear mixed-effect models with random intercepts for participants. Differences between time points were based on estimated marginal means, and effect sizes were standardised using the pretreatment SD. All analyses were conducted in R V.4.5.1.

## Results

### Feasibility

#### Study uptake

Between March and December 2024, 158 families registered interest (table 3). Recruitment was paused during June–August, yielding 7 months of active recruitment. Approximately 23% of enquiries were prompted by a health care-provider recommendation to self-refer to the study. During active months, the mean recruitment rate was 0.8 participants per week.

**Table 3:** Feasibility and acceptability outcomes

Domain	Indicator	Result	Comment
Recruitment period	Active accrual months	March–December 2024 (paused June–August)	Seven months of active recruitment
Registrants	Families registered interest	158	Nationwide sample
Screening	Completed telephone screen	85 (54% of registrants)	
	Completed CAPS-CA-5 assessment	36 (42% of those screened)	
Enrolment	Enrolled participants	22 (61% of those assessed)	No study drop-outs
Accrual rate	Mean enrolment per week	0.8 participants per week	Active months only
Therapists	Number of therapists delivering iTF-CBT	7	Each treated 2–4 cases
Treatment duration	Planned treatment period	12 weeks	Minor deviations (±1–2 weeks due to illness)
Timeliness	Mean time registration → treatment	95.7 days (50.7 excluding summer delay)	
Data completeness	1 month follow-up	21/22 (≈95%) completed	Final assessment 6 May 2025
Resource use	Mean asynchronous messaging time per module	Adolescents=8.90 min (SD 0.25); caregivers=9.44 min (SD 3.46)	
	Mean phone/video calls per recipient	Adolescents=1.91 (SD 3.07); caregivers=1.36 (SD 1.83)	10 adolescents and 13 caregivers had ≥1 call
Recruitment cost	Estimated per participant	≈151 GBP	
Adherence	Modules completed (mean of 12)	Adolescents=10.5 (SD 2.33); caregivers=10.5 (SD 2.48)	50% adolescents and 54.5% caregivers completed all modules
Acceptability	Discontinued treatment	1 adolescent–caregiver dyad (4.5 %)	
Credibility/expectancy	Mean (SD)	Adolescents=34.32 (6.70); caregivers=41.05 (8.62)	Measured using the Credibility/Expectancy Questionnaire (CEQ)
Satisfaction (CSQ-8)	Mean (SD)	Adolescents (n=17) = 24.18 (5.13); caregivers (n=20) = 26.95 (3.25)	Item-level means 2.41–3.53 (adolescents); 2.80–3.85 (caregivers)

Values are presented as mean (SD) unless otherwise stated.

CAPS-CA-5, Clinician-Administered PTSD Scale for Children and Adolescents for DSM-5; CEQ, Credibility/Expectancy Questionnaire; CSQ, Client Satisfaction Questionnaire; iTF-CBT, internet-based trauma-focused cognitive behavioural therapy; PTSD, post-traumatic stress disorder.

**Figure 1:**
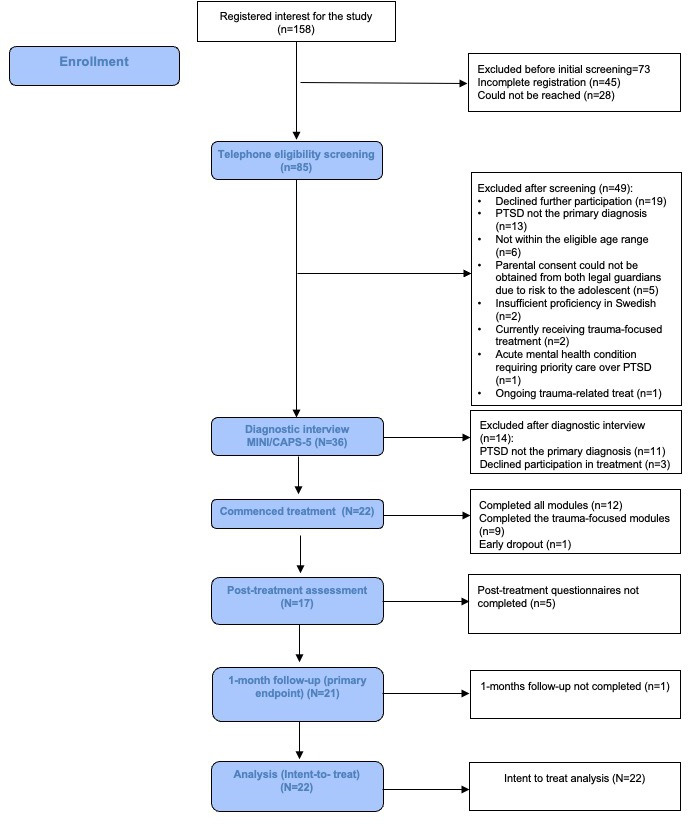
CONSORT flow diagram of participant progress through the study. CAPS-CA-5, Clinician-Administered PTSD Scale for Children and Adolescents for DSM-5; CONSORT, CONsolidated Standards Of Reporting Trial; MINI-KID, Mini International Neuropsychiatric Interview for Children and Adolescents; PTSD, post-traumatic stress disorder.

Of the 158 registrants, 85 adolescents/caregivers (54%) completed a telephone screen and 36 (42% of those screened) completed the CAPS-CA-5 assessment; 22 adolescents from across Sweden were enrolled (61% of those assessed). Baseline demographic and clinical characteristics are summarised in table 1, figure 1 and show participant flow. There were no study drop-outs.

#### Timeliness

The mean time from registration to treatment was 95.7 days. However, a substantial portion of participants experienced a delay due to the Swedish summer break. When excluding this group, the mean registration to treatment time decreased to 50.7 days.

#### Data completeness

At the 1 month follow-up primary endpoint, outcome data were available for 21/22 participants (≈95% completeness). The final 1 month assessment was completed on 6 May 2025.

#### Resource use and cost

Average asynchronous messaging time per module was 8.90 min for adolescents (SD=0.25) and 9.44 min (SD=3.46) min for caregivers. Ten adolescents and 13 caregivers had ≥1 phone or video call; the mean number of calls was 1.91 (SD=3.07) for adolescents and 1.36 for caregivers (SD=1.83). Recruitment costs were estimated at approximately 151 GBP per participant.

### Acceptability of iTF-CBT

#### Treatment adherence

On average, 10.50 of 12 modules were completed by both adolescents (SD=2.33) and caregivers (SD=2.48). By the end of treatment, 11 adolescents (50%) and 12 caregivers (55%) completed all modules. One adolescent–caregiver dyad discontinued treatment.

#### Credibility and satisfaction

Average treatment credibility/expectancy (CEQ) was 34.32 (SD=6.70) for adolescents and 41.05 (SD=8.62) for caregivers. Mean treatment satisfaction (CSQ-8) for adolescents was 24.18 (SD=5.13), indicating a generally high level of satisfaction with the intervention. The corresponding numbers for caregivers were 26.95 (SD=3.25). Item-level means ranged from 2.41 to 3.53 (adolescents) and 2.80 to 3.85 (caregivers) on the 4-point scale, suggesting consistently positive experiences across most satisfaction domains.

#### Negative effects and adverse events

Between baseline and the 1 month follow-up, a total of 196 negative treatment-related effects were reported by both adolescents and caregivers (table 4). Adolescents reported 102 treatment-related events, with a mean impact rating of 1.91 on a 0–4 scale. The most reported event was unpleasant thoughts. Caregivers reported 89 events, with a mean impact rating of 2.12. The most frequent events reported by caregivers were lack of motivation for treatment and unpleasant thoughts.

**Table 4:** Negative effects and adverse events

Category	Definition/example	Adolescents, N (%)	Caregivers, N (%)
Negative effects (NEQ)	Total treatment-related effects reported between baseline and 1 month follow-up (NEQ completers: adolescents n=17) and caregivers=20)	102 events (treatment-related). Mean impact 1.91	89 events (treatment-related). Mean impact 2.12
Most common NEQ items	Unpleasant thoughts	16 (94.1%)	13 (64.7%)
	Lack of motivation for treatment	11 (55.0)	13 (65.0)
Adverse events (weekly self-report)	≥1 adverse event during treatment (open-ended AE item)	9/22 (40.9%)	10/22 (45.5%)
Most frequent AEs	Increased anxiety/tiredness/feeling low or depressed	9.1%	22.7% anxiety, 13.6% low mood
Suicidal ideation	Item≥3 (‘I have thoughts about death and about killing myself’)	1 (4.5%)	–
Serious adverse event (SAE)	Hospital admission	1 (4.5%)	–

Values are numbers (%) unless otherwise stated. Mean impact scores range 0 (‘no impact’) to 4 (‘very severe impact’).

AE, adverse event; NEQ, Negative Effects Questionnaire; SAE, serious adverse event.

Based on the weekly open-ended AEs question, 40.1% of adolescents reported at least one AE (median=0, range 0–7) during treatment. The most common were increased anxiety, fatigue and feeling low or more depressed (9%). Among caregivers, 45% reported at least one AE, most frequently increased anxiety and worry (23%) or feeling low (14%). Suicidal ideation (score≥3 on the item: ‘I have thoughts about death and about killing myself’) was reported by one adolescent. A single SAE occurred (hospital admission), that investigators judged unrelated to the intervention.

Exploratory analyses indicated reductions in clinician-rated PTSD symptoms (CAPS-CA) between baseline and the 1 month follow-up (table 5). The estimated mean change was −12.04 (95% CI −16.2 to −7.83), corresponding to a large within-group effect size (Cohen’s d=1.27).

**Table 5:** Mean scores (SD) on clinician- and self-reported outcome measures at baseline, post-treatment and 1 month follow-up

Measure	M (SD) range
Clinician rated	
CAPS-CA-5	
Baseline (n=22)	34.68 (SD=9.51), 19–52
1 month post (n=21)	23.05 (SD=10.51), 5–40
Self- and caregiver assessed	
CATS-2 child	
Baseline (n=22)	36.64 (SD=4.69), 26–44
Post-treatment (n=17)	28.76 (SD=11.76), 0–42
1 month post (n=17)	29.71 (SD=10.62), 8–47
CATS-2 parent	
Baseline (n=22)	32.41 (5.18), 24–47
Post-treatment (n=20)	20.90 (11.39), 0–50
1 month post (n=20)	19.05 (11.48), 0–42

Values are presented as mean (SD). Higher scores indicate greater symptom severity.

CAPS-CA, Clinician-Administered PTSD Scale for Children and Adolescents; CATS-2-C, Child and Adolescent Trauma Screen (child report); CATS-2-P, Child and Adolescent Trauma Screen (parent report).

At the 1 month follow-up, 10 of the 21 participants (47.6%) who completed the CAPS-CA assessment no longer met diagnostic criteria for PTSD.

Exploratory analyses of other, more secondary outcomes suggested reductions in self-reported PTSD symptoms (CATS-2-C; n=17 with available data at the 1 month follow-up) between baseline and follow-up, although the magnitude of change was smaller than that observed in clinician-rated outcomes. Self-reported post-traumatic stress symptoms (CATS-2-C) decreased between baseline and follow-up, and caregiver reports of post-traumatic stress symptoms (CATS-2-P) showed a comparable reduction (table 5). Improvements were also observed in child-reported well-being (KIDSCREEN-C), whereas caregiver ratings of well-being (KIDSCREEN-P) did not change substantially.

For functional impairment, no clear change was observed in child-reported functioning (WSAS-Y), while caregiver reports (WSAS-P) suggested moderate improvement. No meaningful change was observed in child-reported depressive symptoms (MFQ-C), whereas caregiver reports indicated a reduction in depressive symptoms (MFQ-P) (online supplemental table S1).

## Discussion

This pilot study aimed to evaluate the feasibility, acceptability, safety and preliminary clinical effects of a therapist-guided, iTF-CBT programme for adolescents with diagnosed PTSD, delivered within a non-for-profit specialist clinic for children and young people operated by Save the Children Sweden, with parallel caregiver access. Overall, the intervention appeared feasible and well accepted and associated with promising within-group improvements. Given the uncontrolled feasibility design and small sample size, clinical outcomes should be interpreted as exploratory signals rather than evidence of treatment effectiveness. Retention and adherence were strong; credibility and satisfaction ratings were favourable and no intervention-related SAEs were observed. These findings align with prior evidence for internet-delivered interventions in adult PTSD^9 25^ and extend the paediatric iCBT literature by targeting adolescents with diagnosed PTSD. Recruitment primarily relied on advertising directed at caregivers. While this approach was adequate, it may have been faster and more effective if it had involved direct engagement with youth. Future trials should explore youth-centred outreach strategies such as advertisements directed to the youth instead of caregivers and referrals through healthcare and other services. Despite these barriers, inclusion rates among assessed youth were high. However, recruitment procedures may require optimisation in a future RCT. High treatment adherence and regular therapist contact indicate that asynchronous, message-based guidance with optional calls is feasible for both families and clinicians. All participants screened for at least one additional diagnosis, and about two-thirds screened positive for ADHD or ASD. Despite this high comorbidity and associated dropout risk, over 95% maintained regular therapist contact across 12 weeks, demonstrating strong engagement. As expected in exposure-based care, short-term increases in distress were common but typically transient and treatment-concordant. One SAE occurred but was deemed unrelated to treatment. This underscores the importance of ongoing risk monitoring and predefined escalation pathways in digital PTSD treatment. The digital format was generally well received by both adolescents and caregivers. Average therapist time was approximately 18 min per family per week, substantially lower than the 90 min typically required for face-to-face TF-CBT and similar to earlier iCBT studies.^26^ If confirmed in future trials, this efficiency gain would have clear clinical and health-economic relevance. Within-group analyses showed large clinician-rated improvements and moderate adolescent self-reported improvements, whereas caregivers reported larger reductions across several domains. Such discrepancies between informants are common in the literature on PTSD but also other conditions.^27 28^ They underscore the importance of using multiple informants when evaluating treatment effects in youth PTSD. More participants completed the follow-up CAPS-CA-5 interview than the CATS-2 self-report, indicating lower completion rates for self-report measures. In a future RCT, this may be addressed by allocating dedicated time for participants to complete self-report measures in close connection to the interview, rather than relying on reminders at the end of the session or via text messages. One participant showed symptom worsening (>5 points on CAPS-CA-5) and required stepped-up care. This underscores the need for ongoing monitoring and timely referral to more intensive treatment in future trials. This study has several strengths, including the use of CAPS-CA-5 as the gold-standard outcome, robust safety monitoring and delivery in a non-profit specialist care clinic setting with high retention. Limitations include the lack of a control group, self-selection of participants and provision in Swedish only, limiting generalisability to migrant and refugee populations. Further, improved recruitment pace will be necessary in a future RCT. A further limitation of the study is the absence of explicit a priori progression criteria. Consequently, the evaluation of feasibility is based on exploratory outcomes rather than predefined benchmarks. The lack of predefined progression criteria means that interpretations of feasibility rely on exploratory judgement rather than objective thresholds, which may reduce transparency in determining readiness for a future trial.

## Conclusions

Therapist-guided iTF-CBT for adolescents with PTSD appeared feasible, acceptable and safe in a routine care setting. Exploratory findings suggested potential clinical benefits; however, these results should be interpreted with caution given the nature of the study design. Future RCTs are warranted to establish efficacy, safety, cost-effectiveness and long-term outcomes.

## Supplementary material

10.1136/bmjopen-2026-117024 online supplemental file 1

10.1136/bmjopen-2026-117024 online supplemental file 2

10.1136/bmjopen-2026-117024 online supplemental file 3

## Data Availability

Data are available upon reasonable request.
