# Supplementary material for: Feasibility and acceptability of therapist-guided, asynchronous, internet-delivered trauma-focused CBT for adolescents with PTSD: a single-group feasibility trial in Sweden
Source: BMJ Open. 2026 May 27;16(5):e117024. doi: 10.1136/bmjopen-2026-117024 (PMC13218193; doi:10.1136/bmjopen-2026-117024)
Supplement: online supplemental file 1 [file bmjopen-16-5-s001.pdf]

Vårdnadshavare

Jag samtycker till att mitt barn deltar i studien:

En forskningsstudie om internetförmedlad traumafokuserad KBT Internetförmedlad för ungdomar med PTSD

● Jag har muntligen informerats och tagit del av ovanstående skriftlig information. Jag har blivit informerad om syftet med studien, haft tillfälle att ställa frågor och haft tillräckligt med tid att tänka över mitt beslut.

● Jag samtycker till att mitt barn deltar i forskningsstudien och vet att deltagandet är frivilligt.

● Jag vet att deltagande kan avbrytas när som helst utan att skäl behöver anges och att det inte

påverkar barnets framtida vård och behandling.

● Jag samtycker till datainsamling och vet att patientsekretess gäller.

● Jag samtycker till att ni inhämtar journaluppgifter från andra vårdgivare.

● Jag har förstått att identitet inte kommer avslöjas i sammanställningar av resultaten

Vi godkänner deltagande i studien ” internetförmedlad traumafokuserad KBT ör ungdomar med PTSD” enligt beskrivningen i det här brevet.

Vi godkänner att bedömningssamtal spelas in på ljud i utbildningssyfte för framtida studier.

Ungdomens för- och efternamn:

Vårdnadshavares namnteckning

Vårdnadshavarens namnteckning

Datum

Datum

Namnförtydligande

Namnförtydligande

Jag har förklarat studiens upplägg och syfte för ovanstående vårdnadshavare samt inhämtat samtycke för deltagande

Psykologens namnteckning Datum

Namnförtydligande

Etikprövningsmyndigheten
2023-06185-01-462295
2023-10-12

Ungdom

Jag samtycker till att delta i studien:

En forskningsstudie om Internetförmedlad traumafokuserad KBT behandling för ungdomar med PTSD

- Jag har fått muntlig och skriftlig information om studien.

- Jag har haft möjlighet att ställa frågor.

- Jag vet att det är frivilligt att tacka ja till den här studien.

- Jag vet att jag kan avbryta när jag vill utan att ge en förklaring, och att det inte påverkar mina möjligheter till vård och behandling.

- Jag vet att den här studien samlar in uppgifter om min person och sparar svar på enkäter.

- Jag vet att patientsekretess gäller, det vill säga de jag träffar inom studien har tystnadsplikt och grundregeln är att vi inte berättar något om en patient för andra personer.

- Jag säger ja till att ni hämtar information om vård jag fått i mina sjukvårdsjournaler.

- Jag har förstått att vem jag är hålls hemligt när resultat från forskningen presenteras.

Jag vill delta i studien ” Internetförmedlad traumafokuserad KBT för ungdomar med PTSD”.

Jag godkänner att bedömningssamtal spelas in på ljud så att ny personal kan lära sig att göra bedömning i framtida studier.

Ungdomens namnteckning Datum

Namnförtydligande

Etikprövningsmyndigheten
2023-06185-01-462295
2023-10-12
