# Supplementary material for: Feasibility and acceptability of therapist-guided, asynchronous, internet-delivered trauma-focused CBT for adolescents with PTSD: a single-group feasibility trial in Sweden
Source: BMJ Open. 2026 May 27;16(5):e117024. doi: 10.1136/bmjopen-2026-117024 (PMC13218193; doi:10.1136/bmjopen-2026-117024)
Supplement: online supplemental file 2 [file bmjopen-16-5-s002.pdf]

Etikprövningsmyndigheten
2023-06185-01-466116
2023-10-12

BACKGROUND.......... 4

OBJECTIVE.......... 4

PRIMARY OBJECTIVE.......... 4

DESCRIPTION OF THE STUDY.......... 5

OUTLINE.......... 6

INCLUSION CRITERIA.......... 6
QUALITY CONTROL.......... 6
INTERVENTION.......... 6
COMPARATOR.......... 6
SAMPLE SIZE.......... 7
OUTCOME MEASURES.......... 7

PRIMARY MEASURES.......... 7

ASSESSMENT POINTS.......... 8

OUTCOME MEASURES.......... 8

ASSESSMENTS AND PROCEDURES.......... 9

INCLUSION PROCEDURE.......... 9
INCLUSION.......... 10
BASELINE ASSESSMENT.......... 10
DURING TREATMENT.......... 10
POST ASSESSMENT.......... 10
1-MONTH FOLLOW UP.......... 10
6-MONTH FOLLOW-UP.......... 10
ADVERSE EVENTS.......... 10
SAFETY PROCEDURES.......... 10

ETHICS.......... 11

DATA MANAGEMENT.......... 11

STUDY SITE AND RESEARCH GROUP.......... 11

TIME PLAN.......... 11

PROCEEDINGS FOR ADVERSE EVENTS.......... 12

DEFINITION OF ADVERSE EVENTS.......... 12
DEFINITION OF SERIOUS ADVERSE EVENTS.......... 12
ASSESSMENT OF ADVERSE EVENTS.......... 12
ASSESSMENT OF INTENSITY.......... 12
ASSESSMENT OF CAUSALITY.......... 13
IMPLICATIONS.......... 13

Etikprövningsmyndigheten
2023-06185-01-466116
2023-10-12

REFERENCES.. 14

Etikprövningsmyndigheten
2023-06185-01-466116
2023-10-12

BACKGROUND

The World Health Organization identifies trauma as a major global public health problem. Nearly 60% will experience a potentially traumatic event by the end of adolescence [1]. For example, 40 million children aged below 15 years fall victim to violence each year. The prevalence of post-traumatic stress disorder (PTSD) in children exposed to trauma is estimated to be between 5% to 16% [2,3]. It is even more common in particular populations such as refugee children [4]. PTSD is a debilitating psychiatric condition involving distressing symptoms such as intrusions from the traumatic event, inability to be around trauma reminders, cognitive and mood changes, and arousal symptoms and associated with considerable functional impairment and comorbidity [5]. For children with untreated PTSD, extensive data shows a significantly increased risk for substance use, suicidality, and poorer mental health/functioning [6,7] as well as impaired school performance [8]. TF-CBT has proven effective in treating youth with PTSD and is considered first-line treatment but is rarely available in regular care.

Given the large group of afflicted and the treatment gap, action is clearly needed to increase availability. A possible solution to significantly improve access to evidence-based treatment would be the use of remotely delivered digital intervention. Internet-delivered trauma-focused CBT (iTF-CBT) may also carry several other advantages compared to traditional psychological treatments (eg. less therapist time per patient, bridging geographical distances between therapist and patient, and provision of a standardized intervention). Internet-delivered treatment has shown promising results in treating adults with PTSD [9] and as early intervention after trauma [10]. Furthermore, research has indicated that ICBT is an effective treatment format for children [11]. But, to our knowledge, this project is the first large RCT to evaluate digital iTF-CBT for children with PTSD.

Etikprövningsmyndigheten
2023-06185-01-466116
2023-10-12

OBJECTIVE

PRIMARY OBJECTIVE

There is an urgent need for evidence-based treatments for children with PTSD that can easily be made available to a large number of afflicted. The overall aim of the project is to increase the availability of evidence-based psychological treatments for adolescents with PTSD by developing and evaluating iTF-CBT for this target group. The main objectives are to establish the efficacy, cost-effectiveness, and long-term effects of iTF-CBT for adolescents with PTSD in an RCT (N=140) by comparing guided iTF-CBT with an attention control group consisting of therapist-supported internet-delivered psychoeducation and support.

Research questions:

1. Is guided iTF-CBT more efficacious than an active control condition (therapist-supported internet-delivered psychoeducation and support) in regard to reduction of blinded assessor rated PTSD symptom severity 1-month post-treatment?
2. Is guided iTF-CBT more cost-effective than an active control condition (therapist-supported internet-delivered psychoeducation and support) in regard to reduction of blinded assessor rated PTSD symptom severity 1-month post-treatment?

3. Are the therapeutic gains of iTF-CBT maintained at long term follow up (6 and 12 months after treatment)?
4. How do children, parents and therapists perceive iTF-CBT?

DESCRIPTION OF THE STUDY

This study is a single-blind, parallel-group superiority trial with an internal pilot. Study participants will be randomly assigned to 12 weeks of either iTF-CT or therapist-supported internet-delivered psychoeducation on a 1:1 ratio without restriction. To prevent potential selection bias related to the randomization procedure, we will use an external party, the Karolinska Trial Alliance (KTA, www.karolinskatrialalliance.se) for that. Allocation concealment will be ensured through randomization after the decision to include each participant has been made. Evaluation includes efficacy, cost-effectiveness, and long-term effects. Outcome measures will be evaluated at baseline, post-treatment, 1-months (primary endpoint), and 6- and 12-months follow-up by assessors blinded to treatment allocation. To ensure blinding integrity, participants and their guardians will receive explicit instructions not to disclose which treatment they have received. After completing the assessment, the blind raters guess the participant's group allocation. If participants inadvertently disclose the treatment allocation during the assessment, another blind assessor will re-assess the diagnosis based on recordings of the clinical interviews. Participants will be recruited nationally through self- or clinician referral. Self-referral will be made available through a dedicated secure website.

Etikprövningsmyndigheten
2023-06185-01-466116
2023-10-12

The first 20 included participants will not be randomized but will be part of an internal pilot. They will be asked to participate in process evaluation interviews after treatment completion. Interviews will also be conducted with therapists and clinicians recruiting to the study and providing treatment. The aim of this is to see that the recruitment and treatment procedures are feasible and acceptable and that retention to the trial are sufficient to allow the trial to progress.

Consecutive applicants referred to the study are assessed for eligibility. Interested applicants are screened on the telephone after completion of screening measures for PTSD, and eligible youths and their families are invited to a psychiatric assessment via video-conferencing or face-to-face. The aim of this visit is ensuring eligibility by verification that PTSD is the primary diagnosis and other inclusion/exclusion criteria, assess severity of PTSD symptoms, and assess for comorbidity. The gold standard clinical interview CAPS-5-CA[12] that assesses past month diagnosis of PTSD and PTSD symptom severity will be used to ensure validity and reliability of the PTSD diagnosis. The interview will also include the child version of the Mini International Neuropsychiatric Interview (MINI-KID) [13]. The assessor also provides detailed information about the study and treatment including objectives, benefits, risks, and requirements imposed by the study during this visit. The information is also provided in writing. If the applicant is eligible for the study, the study participant and guardian sign informed consent digitally during this visit.

OUTLINE

INCLUSION CRITERIA

Inclusion criteria

- 13-17 years,
- Primary PTSD diagnosis
- Fluent in Swedish
- Access to the Internet at home or can use vouchers for internet-access to their phone.
- Parent/guardian willing and able to take part in treatment.

Exclusion criteria:
PTSD is not the primary concern

- Initiation or adjustment of any psychotropic medication within the last 4 weeks prior to commencement of treatment
- Serious mental health symptoms, such as mania, psychosis, alcohol, or substance use disorders or current suicide risk warranting immediate clinical attention.
- Ongoing trauma-focused CBT or Eye movement desensitization and reprocessing Therapy
- Ongoing trauma-related threat

Etikprövningsmyndigheten
2023-06185-01-466116
2023-10-12

QUALITY CONTROL

The trial will follow Good Clinical Practice (GCP).

INTERVENTION

The iTF-CBT protocol mirrors the exact content of first-line treatment for children with PTSD but is delivered through a digital platform and will comprise of text-based material as well as audio files and video illustrations in an age-appropriate manner that the participant gains access to sequentially. iTF-CBT will be therapist-assisted, and each participant will have a designated therapist to guide them through treatment. Treatment will span over 12 weeks and integrates cognitive, behavioral, interpersonal, and family therapy principles as well as trauma interventions. Parallel to the child's treatment, the caregivers are provided the same components and parenting practices, and joint child-parent activities are included.

COMPARATOR

The active control will consist of therapist-supported internet-delivered psychoeducation and support for the same amount of time. The idea behind this intervention is to control for general non-specific factors (i.e., placebo effects) and at the same time does not exaggerate apparent efficacy of ITF-CBT which may be the case if using a wait-list control condition.

Sample size

Outcome measures

The power analysis for the RCT is based on the gold-standard interview CAPS-5 where a clinically significant difference in treatment effects is 10 points, which is the between groups difference we aim to detect. With a standard deviation of the CAPS-5 to be 20, this represents a standardized mean difference in terms of Cohens *d* of 0.5 and to use it conservatively we used Cohens *d* 0.4 in our calculations. The estimated number needed is then N=240 (moderate between-group effect size against active control condition at 1-month post-treatment; 95% power, 10% data attrition rate, and an α level of 0.04). All analysis will be conducted according to the intent to treat principle and we will follow the CONSORT guidelines in reporting and analyzing the data.

The primary hypothesis (clinician-rated CAPS-5-CA) will be evaluated using a linear mixed-effects model with fixed effects of time and group, their interaction, as well as random intercept and slope. Treatment response will be defined as at least 6 points improvement on the CAPS-5 between baseline and participants' last available measurement between baseline and 12-month follow-up (adapted from Schnurr & Lunney, 2016). Secondary self-rated outcomes will be estimated using linear mixed-effects models with repeated measurements and fixed effects of time and group, their interaction, as well as a random intercept and slope.

Etikprövningsmyndigheten
2023-06185-01-466116
2023-10-12

We will follow the Consolidated Health Economic Evaluation Reporting Standards (CHEERS) guidelines to report the health economic evaluation and look at both direct costs (e.g., therapist time per patient) and other societal costs (e.g., absence from school, caregivers' absence from work). Cost differences between iTF-CBT and the active control condition will be calculated, as well as the incremental cost-effectiveness ratio (ICER). The ICER is a global estimate of cost-effectiveness and represents the additional cost that is associated with the additional clinical effect in one intervention compared with another. Intra-Class Correlations will be used to calculate Interrater agreement on the CAPS-5-CA.

PRIMARY MEASURES

Feasibility will be assessed by evaluating the study procedures and provision of the treatment using the RE-AIM framework [14], focusing on the intervention's reach (percentage eligible/excluded, reasons for refusal or exclusion), effectiveness (symptom reduction and impact on quality of life), adoption (attrition, adherence, client satisfaction), implementation (clinician resources), and maintenance (effects on symptom measures and measures of quality of life at the 6-month follow-up).

Treatment acceptability will be defined in accordance to the definition by Sekhon et al. (2017)[15]: "the extent to which people delivering or receiving a healthcare intervention consider it to be appropriate, based on anticipated or experienced cognitive and emotional responses to the intervention". This definition considers acceptability to consist of seven component constructs: affective attitude, burden, perceived effectiveness, ethicality,

intervention coherence, opportunity costs, and self-efficacy. We consider the proportion of participants that go through the entire treatment period, adverse events related to the treatment, number of dropouts and number of individuals offered the intervention but declined part of treatment acceptability. Qualitative interviews will be conducted and scheduled separately post-treatment with a focus on acceptability. Client Satisfaction Questionnaire (CSQ-8) will be used to assess patient satisfaction with treatment [16]. Data on adverse events will be collected each treatment week using a standardized checklist (Safety Monitoring Uniform Report Form).

The primary outcome measure will be CAPS-5-CA, the gold standard clinical interview for PTSD that assess PTSD symptom severity and past month diagnosis of PTSD. CAPS-5-CA assesses the 20 DSM-5 PTSD symptoms, the onset and duration of symptoms, subjective distress, impact of symptoms on social and occupational functioning, improvement in symptoms since a previous CAPS administration, overall response validity, overall PTSD severity, and specifications for the dissociative subtype of PTSD (depersonalization and derealization).

Secondary outcome measures are self-rated PTSD symptom severity, other mental health problems, clinician-rated symptom improvement, global functioning, health-related quality of life, cost data, therapeutic alliance, treatment credibility, client satisfaction, adverse events.

Etikprövningsmyndigheten
2023-06185-01-466116
2023-10-12

Assessment points

The primary outcome measure, CAPS-5 CA, will be conducted at baseline, 1-month post-treatment (primary endpoint), and at 6-month and 12-month follow-ups (secondary endpoints). Self-administered measures will be used at baseline, post-treatment, 1-month post-treatment (primary endpoint), and at 6-month and 12-month follow-ups. Self-administered measures of PTSD (CATS-2) and adverse events are also administered weekly through the treatment period.

Outcome measures

- Clinician Administered PTSD Scale Children and Adolescent version 5 (CAPS-5-CA)[12], the gold standard clinical interview to assess PTSD symptom severity and diagnosis of PTSD [42] at baseline and the 1-month, 6-months and 12-months follow up.
- The Child and Adolescent Trauma Screen (CATS-2)[17] at baseline, weekly through the treatment period, post-treatment and at the 1-month, 6-month and 12-month follow up.
- Mood and Feelings Questionnaire (MFQ)[18] at baseline, post-treatment and at the 1-month, 6-month and 12-month follow up.

- The Education, Work and Social Adjustment Scale, Youth and Parent (EWSAS)[19] at baseline, post-treatment and at the 1-month, 6-month and 12-month follow up.
- Treatment Inventory of Costs in Psychiatric Patients (TIC-P) [20]at baseline, post-treatment and at the 1-month, 6-month and 12-month follow up.
- The Negative Effects Questionnaire (NEQ)[21] at post-treatment and at the 1-month, 6-month and 12-month follow up.
- Assessment of Quality of Life KIDSCREEN at baseline, post-treatment and at the 1-month, 6-month and 12-month follow up.
- The Client Satisfaction Questionnaire (CSQ-8)[22] at post-treatment.
- The Working Alliance Inventory (WAI)[23] after completion of treatment module 3.
- The Credibility/Expectancy Questionnaire (CEQ)[24] after completion of treatment module 1.

ASSESSMENTS AND PROCEDURES

Etikprövningsmyndigheten
2023-06185-01-466116
2023-10-12

INCLUSION PROCEDURE

Participants will be recruited through the designated website for the study. Information about the study will be made available at psychiatric clinics and advertisements in social media and newspapers about the study. All participants will during the study period be patients at BUP Internetenhet or Save the Children.

In the first stage of recruitment to the study, interested applicants do an Internet-administered screening on an encrypted webpage using questions about inclusion and exclusion criteria, demographics, symptoms of post-traumatic stress, depressive symptoms, quality of life and alcohol and drug consumption. Written information about the study will be given before the screening including objectives, benefits, risks and requirements imposed by the study. The applicant is also provided with contact information to the study personnel and have the possibility to call and ask for more details or clarifications before signing informed consent in the digital platform.

Interested applicants are screened on the telephone, and preliminary eligible youths and their families are invited to a psychiatric assessment via video-conferencing or face-to-face. The aim of this visit is ensuring eligibility by verification that PTSD is the primary diagnosis and other inclusion/exclusion criteria, assess severity of PTSD symptoms, and assess for comorbidity. The gold standard clinical interview CAPS-5-CA that assesses past month diagnosis of PTSD and PTSD symptom severity will be used to ensure validity and reliability of the PTSD diagnosis. The interview will also include the child version of the Mini International Neuropsychiatric Interview version 7 (MINI-KID) [13]. According to clinical routines, a suicide-risk assessment is also mandatory.

Inclusion

Decisions on inclusion are taken by a licensed psychologist and the principal investigator (Maria Bragesjö) together.

Baseline assessment

Before the start of treatment, participants will conduct baseline assessments consisting of CATS-2, MFQ, WSAS, KIDSCREEN and TIC-P.

during treatment

Weekly during the treatment period, CATS-2, a question about suicidal thoughts and questions about adverse events are administered.

Post assessment

Post-assessment is done using the same outcome measure as for the baseline assessment but with the addition of a self-rating questionnaire about acceptability and satisfaction with the treatment, Client Satisfaction Questionnaire (CSQ-8).[16] Interviews with the participants and their caregivers will be conducted after completion of treatment.

Etikprövningsmyndigheten
2023-06185-01-466116
2023-10-12

1-month follow up

The same self-rated assessment questionnaires as at baseline will be used with the addition of the CAPS-5-CA interview. Qualitative interviews will also be conducted and scheduled separately. The assessment will include the need for further psychiatric care.

6-month Follow-up

Long-term follow-up assessments will be conducted at 6 months after completed treatment using the CAPS-5-CA and the same self-rated assessment questionnaires as baseline.

Adverse events

Adverse events data will be collected every week during the treatment period and at each assessment point using a self-rating questionnaire with a free text option where participant is instructed to describe the possible event in detail. If the participant reported any adverse event, additional follow-up questions about intensity, duration etc. will also be given and assessed by the research team and designated therapist. Eventual adverse events will be handled in accordance with good clinical practice.

Safety procedures

Study participants will be able to report the occurrence of possible adverse events or undesirable treatment effects at any point through the study period either through the message function on the Internet platform or by contacting the study personnel by phone. A structured self-report questionnaire to capture the frequency and nature of possible adverse events will be used weekly through treatment and at all follow-up points. The participants

are asked to report and rate the short- and long-term discomfort of any adverse event on a scale from 0 (“did not affect me at all”) to 3 (“affected me very negatively”). Reported incidents will be categorized depending on severity and frequency in line with GCP and will be crosschecked by KTA. All participants will be monitored by the study personnel throughout the study period to ensure participant safety. Written routines for situations of potential risk are made available to assessors and therapists. Serious adverse events (i.e. suicide attempts, serious violent incidents, or admission to hospital) will be documented and monitored by KTA. In case a participant’s symptoms worsen during treatment, the therapist will carry out an immediate in-depth assessment of the symptoms and discuss this with the principal investigator for necessary actions.

ETHICS

The study protocol and necessary documentation will be submitted to the Swedish Ethical Review Authority and must be approved for the study to commence.

DATA MANAGEMENT

Case report form (CRF) data will be imputed to a data file. Statistical analysis will be performed by the research group and quality control and crosscheck will be performed by independent party. Data analysis will be done according to the intention to treat principle. The qualitative interviews will be analyzed using thematic analysis.

Etikprövningsmyndigheten
2023-06185-01-466116
2023-10-12

STUDY SITE AND RESEARCH GROUP

The study is conducted by researchers at Karolinska Institutet, in collaboration with Region Stockholm, BUP Internetenhet and Save The Children. PhD Maria Bragesjö, Karolinska Institutet, is the principal investigator of the study. Professors Eva Serlachius and David Mataix-Cols, associate professor Erik Andersson, PhD Sarah Vigerland and PhD-student Erica Mattelin comprise the rest of the research team.

This proposal is built upon a need for an increase in treatment availability that has been emphasized by policymakers, parents, and children. Adolescents, parents, and experienced clinicians will be involved in the iterative development of the treatment program using a patient and public involvement (PPI) framework to involve children, parents, and stakeholders. This need to involve children and young people has been emphasized in earlier research and is also in line with the Convention of the Rights of the Child, article 12.

TIME PLAN

Participants will be recruited and provided treatment starting winter 2023. Statistical analyses will be done immediately after the last participant has completed the post-

assessment. First publication is estimated to late 2024 or beginning of 2025. All data will be handled and eventually archived according to Swedish and European legislation.

2023 Q3-Q4: Develop the iTF-CBT treatment and set up the platform.
2024: Q1 Commencement of the RCT with an internal pilot study and follow-up assessments. Refinement of treatment.
2024 Q2-Q4: Completion of treatments and 6 months follow-up assessments. Analysis of results, manuscript draft of pilot part of RCT.
2026 Q4: Completion of 12 months follow-up assessments.

PROCEEDINGS FOR ADVERSE EVENTS

DEFINITION OF ADVERSE EVENTS

An Adverse Event (AE) is any untoward in a subject receiving an intervention which does not necessarily have a causal relationship with the intervention. An AE can be any unfavorable and unintended sign, symptom or disease temporally associated with receiving the intervention, whether or not related to it.

Etikprövningsmyndigheten
2023-06185-01-466116
2023-10-12

DEFINITION OF SERIOUS ADVERSE EVENTS

Each AE is to be classified by the investigator as serious or non-serious. Seriousness is not defined by a medical term; it is a result or an outcome. An AE is defined as a Serious Adverse Event (SAE) if it:

- results in death
- is life-threatening
- requires inpatient hospitalization or prolongation of existing hospitalization
- results in persistent or significant disability/incapacity

ASSESSMENT OF ADVERSE EVENTS

AE is assessed in the treatment platform including an open question.

ASSESSMENT OF INTENSITY

Each AE is to be classified by the investigator as mild, moderate or severe.

Mild: Acceptable. The subject is aware of symptoms or signs, but they are easy tolerated.

Moderate: Disturbing. The AE is discomfort enough to interfere with usual daily activity.

Severe: Unacceptable. The subject is incapable to work or to do usual daily activities.

ASSESSMENT OF CAUSALITY

Unlikely: The event is most likely related to an aetiology other than the treatment.

Possible: A causal relationship is conceivable and cannot be dismissed.

Probably: Good reason and sufficient documentation to assume a causal relationship.

IMPLICATIONS

According to a recent study, only 21 % of the 132 reviewed articles on RCTs with psychological interventions reported adverse events (AE)[25]. Furthermore, Rief, Nestoriuc, von Lilienfeld-Toal, Dogan, Schreiber, Hofmann, Barsky and Avorn [26] found that systematic assessment of AE led to a higher frequency of reported AE than less systematic ones, i.e. spontaneous reports. The probability for an AE to be reported was twice as high with structured assessment methods [28]. Hence, the results from the collected AE data in this trial has the potential to provide important information for the scientific community as well as for patients and health care providers.

Etikprövningsmyndigheten
2023-06185-01-466116
2023-10-12

REFERENCES

1. McLaughlin KA, Koenen KC, Hill ED, et al. Trauma exposure and posttraumatic stress disorder in a national sample of adolescents. *Journal of the American Academy of Child & Adolescent Psychiatry*. 2013;52(8):815-830. e14.
2. Merikangas KR, He J-p, Burstein M, et al. Lifetime prevalence of mental disorders in US adolescents: results from the National Comorbidity Survey Replication–Adolescent Supplement (NCS-A). *Journal of the American Academy of Child & Adolescent Psychiatry*. 2010;49(10):980-989.
3. Alisic E, Zalta AK, Van Wesel F, et al. Rates of post-traumatic stress disorder in trauma-exposed children and adolescents: meta-analysis. *The British Journal of Psychiatry*. 2014;204(5):335-340.
4. Blackmore R, Boyle JA, Fazel M, et al. The prevalence of mental illness in refugees and asylum seekers: A systematic review and meta-analysis. *PLoS Med*. Sep 2020;17(9):e1003337. doi:10.1371/journal.pmed.1003337
5. Kessler RC, Petukhova M, Sampson NA, Zaslavsky AM, Wittchen HU. Twelve-month and lifetime prevalence and lifetime morbid risk of anxiety and mood disorders in the United States. *International journal of methods in psychiatric research*. 2012;21(3):169-184.
6. Chapman JF, Ford JD. Relationships between suicide risk, traumatic experiences, and substance use among juvenile detainees. *Archives of Suicide Research*. 2008;12(1):50-61.
7. Jaycox LH, Ebener P, Damesek L, Becker K. Trauma exposure and retention in adolescent substance abuse treatment. *Journal of Traumatic Stress: Official Publication of The International Society for Traumatic Stress Studies*. 2004;17(2):113-121.
8. Vilaplana-Pérez A, Sidorchuk A, Pérez-Vigil A, et al. Assessment of Posttraumatic Stress Disorder and Educational Achievement in Sweden. *JAMA network open*. 2020;3(12):e2028477-e2028477. doi:10.1001/jamanetworkopen.2020.28477
9. Simon N, McGillivray L, Roberts NP, Barawi K, Lewis CE, Bisson JI. Acceptability of internet-based cognitive behavioural therapy (i-CBT) for post-traumatic stress disorder (PTSD): a systematic review. *European journal of psychotraumatology*. 2019;10(1):1646092-1646092. doi:10.1080/20008198.2019.1646092
10. Bragesjö M, Arnberg FK, Olofsdotter Lauri K, Aspvall K, Särnholm J, Andersson E. Condensed Internet-delivered prolonged exposure provided soon after trauma: a randomised trial. *Psychological Medicine*. 2021:1-10. doi:10.1017/S0033291721003706
11. Vigerland S, Lenhard F, Bonnert M, et al. Internet-delivered cognitive behavior therapy for children and adolescents: A systematic review and meta-analysis. *Clinical Psychology Review*. 2016/12/01/ 2016;50:1-10. doi:https://doi.org/10.1016/j.cpr.2016.09.005
12. Pynoos RS, Weathers, F. W., Steinberg, A. M., Marx, B. P., Layne, C. M., Kaloupek, D. G., Schnurr, P. P., Keane, T. M., Blake, D. D., Newman, E., Nader, K. O., & Kriegler, J. A. . *Clinician-Administered PTSD Scale for DSM-5 - Child/Adolescent Version.* . 2015.
13. Sheehan DV, Sheehan KH, Shytle RD, et al. Reliability and validity of the Mini International Neuropsychiatric Interview for Children and Adolescents (MINI-KID). *J Clin Psychiatry*. Mar 2010;71(3):313-26. doi:10.4088/JCP.09m05305whi
14. Glasgow RE, McKay HG, Piette JD, Reynolds KD. The RE-AIM framework for evaluating interventions: what can it tell us about approaches to chronic illness management? *Patient Educ Couns*. Aug 2001;44(2):119-27. doi:10.1016/s0738-3991(00)00186-5
15. Sekhon M, Cartwright M, Francis JJ. Acceptability of healthcare interventions: an overview of reviews and development of a theoretical framework. *BMC Health Services Research*. 2017/01/26 2017;17(1):88. doi:10.1186/s12913-017-2031-8
16. Larsen DL, Attkisson CC, Hargreaves WA, Nguyen TD. Assessment of client/patient satisfaction: Development of a general scale. *Evaluation and Program Planning*. 1979/01/01/ 1979;2(3):197-207. doi:https://doi.org/10.1016/0149-7189(79)90094-6
17. Sachser C, Berliner L, Risch E, et al. The child and Adolescent Trauma Screen 2 (CATS-2) - validation of an instrument to measure DSM-5 and ICD-11 PTSD and complex PTSD in children and adolescents. *Eur J Psychotraumatol*. 2022;13(2):2105580. doi:10.1080/20008066.2022.2105580
18. Development of a short questionnaire for use in epidemiological studies of depression in children and adolescents. John Wiley & Sons; 1995.
19. Mundt JC, Marks IM, Shear MK, Greist JM. The Work and Social Adjustment Scale: a simple measure of impairment in functioning. *British Journal of Psychiatry*. 2002;180(5):461-464. doi:10.1192/bjp.180.5.461

Etikprövningsmyndigheten
2023-06185-01-466116
2023-10-12

20. Bouwmans C, De Jong K, Timman R, et al. Feasibility, reliability and validity of a questionnaire on healthcare consumption and productivity loss in patients with a psychiatric disorder (TiC-P). *BMC Health Serv Res*. Jun 15 2013;13:217. doi:10.1186/1472-6963-13-217
21. Rozental A, Kottorp A, Forsström D, et al. The Negative Effects Questionnaire: psychometric properties of an instrument for assessing negative effects in psychological treatments. *Behav Cogn Psychother*. Sep 2019;47(5):559-572. doi:10.1017/s1352465819000018
22. Larsen DL, Attkisson CC, Hargreaves WA, Nguyen TD. Assessment of client/patient satisfaction: development of a general scale. *Eval Program Plann*. 1979;2(3):197-207. doi:10.1016/0149-7189(79)90094-6
23. Development and validation of the Working Alliance Inventory, 36 American Psychological Association 223-233 (1989).
24. Devilly GJ, Borkovec TD. Psychometric properties of the credibility/expectancy questionnaire. *J Behav Ther Exp Psychiatry*. Jun 2000;31(2):73-86. doi:10.1016/s0005-7916(00)00012-4
25. Jonsson U, Alaie I, Parling T, Arnberg FK. Reporting of harms in randomized controlled trials of psychological interventions for mental and behavioral disorders: a review of current practice. *Contemp Clin Trials*. May 2014;38(1):1-8. doi:10.1016/j.cct.2014.02.005
26. Rief W, Nestoriuc Y, von Lilienfeld-Toal A, et al. Differences in adverse effect reporting in placebo groups in SSRI and tricyclic antidepressant trials: a systematic review and meta-analysis. Meta-Analysis

Research Support, Non-U.S. Gov't

Review. *Drug Saf*. 2009;32(11):1041-56. doi:10.2165/11316580-000000000-00000

Etikprövningsmyndigheten
2023-06185-01-466116
2023-10-12
