# Supplementary material for: Feasibility and acceptability of therapist-guided, asynchronous, internet-delivered trauma-focused CBT for adolescents with PTSD: a single-group feasibility trial in Sweden
Source: BMJ Open. 2026 May 27;16(5):e117024. doi: 10.1136/bmjopen-2026-117024 (PMC13218193; doi:10.1136/bmjopen-2026-117024)
Supplement: Supplementary file 4 [file bmjopen-2026-117024.reviewer_comments.pdf]

PEER REVIEW HISTORY

BMJ Open publishes all reviews undertaken for accepted manuscripts. Reviewers are asked to complete a checklist review form and are provided with free text boxes to elaborate on their assessment. These free text comments are reproduced below.

ARTICLE DETAILS

Title (Provisional)

Feasibility and acceptability of therapist-guided, asynchronous, internet-delivered trauma-focused CBT for adolescents with PTSD: a single-group feasibility trial in Sweden

Authors

Mattelin, Erica; Weyler, Hanna; Andersson, Rebecca; Paulsen, Josefine; Tielman, Sandra; Vikgren, Anna; Bondjers, Kristina; Serlachius , Eva; Mataix-Cols, David; Bragesjo, Maria

---

VERSION 1 - REVIEW

**Reviewer**	**1**
**Name**	**Richards, David**
**Affiliation**	**University of Exeter, Institute for Health Research**
**Date**	**06-Feb-2026**
**COI**	**None**

This study is written as a feasibility study, but the authors fall into the common trap of assigning importance to their clinical outcomes, well beyond what they should do; in effect treating the analysis as if it was a fully powered controlled study. The balance should be tilted more towards feasibility, including progression criteria. I have provided detailed suggestions on this major point and others below.

p5, L39. Uncontrolled within group effect sizes cannot be used to "inform...power calculations" for an RCT. They can only be used to acknowledge whether or not an intervention can be considered as a candidate for future trials. Undoubtedly they will significantly over-estimate effect sizes and the variance around the mean will be too large for any meaningful inferences from such a small population. Please rewrite more conservatively.

P5, L55. Unclear what definition on "1 month" means. Please clarify here that you mean 1 month post the end of treatment. Also, this text would be better moved to the section on measures. It does not belong here.

P6, L6: ethics should be moved to the separate heading on P10

P8, L37: intervention description. It is not very clear how long the intervention lasts. Please provide more clarity here.

P8, L49 "vivo" should be "in-vivo"

P9, L23. It is not true to say that blinding was not feasible because of the non-randomised design. Randomisation is irrelevant. It is more correct to state it is an uncontrolled design.

P9, L26. The section on PPI is inadequate. Far more detail is required to quantify and qualify PPI activity.

P10. Statistical methods. The biggest flaw in the paper is the lack of any mention of progression criteria, nor their analysis, ordinarily recommended using a quantified traffic light system. Why was this approach, recommended by most feasibility guidance, not undertaken? Mellor et al's paper is instructive here: 2023 Apr 15;9(1):59. doi: 10.1186/s40814-023-01291-5. See also my comments on the analysis of clinical outcomes below.

P11, L46 onwards. The attention given to "clinical outcomes" here is overdone. A tiny uncontrolled feasibility study cannot be analysed to this depth against clinical outcomes. Effect sizes are irrelevant other than to show that the intervention COULD be a candidate for further investigation using better designs. My advice would be to reduce this section of the results very significantly and merely use the post/follow up data to provide an indication of the potential of the treatment. Of more use to the reader and future trial funder would be to know how the end point results compare against a clinical and non clinical population. How many of these participants are outside clinically significant parameters at treatment completion, for example? How many still meet diagnostic criteria? The authors are over-analysing and over-promising from their clinical outcomes data.

I also note that the participant assessed (CATS - 2 Child) results are far less impressive than clinician rated measures and only 17/22 participants provided post/follow up measures. PROMS of this nature are the gold standard. I find it hard to believe the difference between baseline and post/FU was as large as the authors state in the results from the table. Once again, what do the post/FU measures mean in terms of post-treatment psychopathology compared to a standard population? Why is level of follow up considered to be so wonderful? It is actually a fairly significant loss of end point data.

P12: discussion. It is very difficult to justify the strong statements on outcomes used by the authors without any progression criteria being specified. Against what criteria is the intervention "feasible and well accepted"?` The same critique applies to the first part of the subsequent para (L50 onwards). These are strong statements pulled out of the air with no a priori criteria specified against which to make these assertions. This is a tiny study, uncontrolled, from a volunteer population with less than 15% of people initially expressing interest taken into the study. The authors overstate their case very much.

In Table 2 the description of initiating traumatic events includes highly specific ones and general descriptions such as bullying. Surely there is a lot of overlap here. Of course, the initiating event is less important (apart from as a focus of treatment) than the clinical presentation, but nonetheless I will not be the only reader who found this list arbitrary.

Reviewer **2**

Name **Hammerle, Florian**

Affiliation **University Medical Center of the Johannes Gutenberg University Mainz, Klinik und Poliklinik für Kinder- und Jugendpsychiatrie und -psychotherapie**

Date **12-Feb-2026**

COI **None**

Thank you for the opportunity to review the manuscript " Therapist-guided internet delivered trauma-focused CBT for adolescents with PTSD: A feasibility trial". The manuscript is of interest and I have great respect for the commitment that went into the study.

Nevertheless, I have a lot of major concerns and concerns about the manuscript:

A main issue is that some portions of the manuscript are misleading. If I would only read the title/abstract, I would expect that the therapy was delivered synchron, but the main the treatment was asynchron (and in large portions not asynchrony but only processed by patients/caregivers/legal guardians themselves) and via a messaging system (neither voice by call nor video). As this seems very important, it should be pointed out throughout the manuscript (and in title/abstract) and the manuscript should be re-written according to this.

E.g. in the introduction (page 5, lines 21-30) treatment efficacy of internet delivered CBT is referred to a meta-analysis which was based on synchron treatment, but I could point a lot of other parts of the manuscript, where these inconsistencies are reported.

This lead to a second major issue: some portions of the manuscript are not carefully elaborated and written. E.g. the before mentioned argument, that internet-based therapy may be effective is based on three references:

11. Vigerland S, Lenhard F, Bonnert M, et al. Internet-delivered cognitive behavior therapy for children and adolescents: A systematic review and meta-analysis. Clinical Psychology Review. 2016/12/01/ 2016;50:1-10. doi:https://doi.org/10.1016/j.cpr.2016.09.005

12. Pynoos RS, Weathers, F. W., Steinberg, A. M., Marx, B. P., Layne, C. M., Kaloupek, D. G., Schnurr, P. P., Keane, T. M., Blake, D. D., Newman, E., Nader, K. O., & Kriegler, J. A. . Clinician-Administered PTSD Scale for DSM-5 - Child/Adolescent Version. . 2015.

13. Sheehan DV, Sheehan KH, Shytle RD, et al. Reliability and validity of the Mini International Neuropsychiatric Interview for Children and Adolescents (MINI-KID). J Clin Psychiatry. Mar 2010;71(3):313-26. doi:10.4088/JCP.09m05305whi

But only the first reference refers to a meta analysis and citation 12 and 13 refer to instruments.

This is also true for terminology. In the abstract, participants are described as „adolescents" but in the strengths and limitations the first strength is stated as "TF-CBT trial for young people". In the Methods' section, under study design, you refer to "Save the Children Sweden" (which I understand is a proper noun) but go on describing clients as "children" (which is another key term than adolescents, young people etc.) and under "Participants" you also use the term "both children and parents" [whereas I am not shure, if all were in fact parents or caregivers/legal guardians]. This is a major inconsistency throughout the manuscript (e.g. also in acceptability and satisfaction "children", safety "adolescents" and elsewhere). I would recommend to use a stringent wording (e.g. stick to the term "adolescents" throughout the manuscript.

As another, but not exclusively, example of inconsistencies, in the "Study, design and setting", aspects of the sample/measures (Independent evaluators administered the CAPS-CA-5 at baseline and at 1-month, the prespecified primary endpoint.) are described, which should be moved to other sections of the manuscript (as they are neither study design nor setting).

This is also true for AE and especially SAE. You report AE and SAE in the results but do not inform in the method how they were measured and especially the categorization as SAE is not included in the method. Skipping your definition and the measurement of SAE (I expect suicide attempt/death, hospital admission >2 days but probably not birth anomaly as I should not be expected to be triggered by psychotherapy etc.) is not acceptable in a manuscript (especially when dealing with participants with PTSD and higher risk for SAE) and must be augmented.

Therefore, the manuscript must be checked thoroughly for a clear red thread and consistency.

Additionally, I have several concerns in regard to the manuscript:

Abstract:

I don't really find the strength that the "intervention was developed in close collaboration with clinicians" really important (whereas the participation of young people [please refer to my before mentioned issue of key terms] could be) because most interventions are developed in close collaboration to clinicians. I would rather recommend use the independency of treatment deliverance and assessors or other aspects.

Introduction:

The first paragraph of the introduction is poorly designed. The first sentence is misleading as the two cited papers [which are somewhat old] don’t report point prevalence rates, but lifetime prevalence. Therefore, violence and traumatic events are not only estimated in the two papers within adolescence but in adolescents, who may have experienced trauma as children. You switch between these prevalence rates of trauma in adolescents, refer to prevalence of PTSD in children and use a citation, which refers to mental illness in refugees to point out an increasing number of community shootings. If I search Wikipedia for community shootings in Sweden, I get this list:

10 January 2004 Knutby, Uppland

1 1 Knutby murder: Alexandra Fossmo was killed. Her employer, 30-year-old IT entrepreneur Daniel Linde, was shot in the head and chest, but survived.[8][9]

18 March 2015 Gothenburg

2 8+ 2015 Gothenburg pub shooting: A gang-related shooting occurred in which a 25-year-old said to be a leading figure in a local gang and a 20-year-old bystander were killed.

2 August 2020 Norsborg, Botkyrka

1 0 Murder of Adriana Naghei Ostrowska: A 12-year-old girl named Adriana was shot dead in an attempted mass shooting.[10]

10 April 2024 Skärholmen, Stockholm

1 0 Murder of Mikael Janicki: A 39-year-old man named Mikael Janicki was shot dead in a pedestrian tunnel in front of his 12-year-old son after confronting a group of youths in Skärholmen, Stockholm.[11]

4 February 2025 Örebro, Närke

11[b]

6 Örebro school shooting: 35-year-old Rickard Andersson shot and killed 10 students and injured 12 others before committing suicide.[12]

29 April 2025 Uppsala, Uppland

3 0 2025 Uppsala shooting: Three people were killed on the eve of the Walpurgis festival.[13] A 16-year-old male suspect was taken into custody.

Although I understand, that there is an increase in community shootings, the absolute prevalence of community shootings is very low and I am in doubt if these isolated shootings (as bad as they are and I am sorry for every dead/harmed person and the relatives/friends) are the main cause of PTSD or a large increase in numbers of PTSD in Sweden.

Even more, it does not seem necessary to point out, that there might be an increase in PTSD prevalence rates as the prevalence rates are already high enough and Sweden is a country with a relatively small population and large distances between many people. Together with

the skepticism of adolescents with PTSD in regard to therapy (e.g. due to shame and guilt) this may be enough to argue for a TF-CBT delivered by internet.

All in all, the complete first paragraph should be redesigned to follow a better red thread.

Participants: how did you evaluate the suicide risk? Did you use any kind of interview (such as the C-SSRS)? If not, this should be described and mentioned as limitation.

There is no record of trauma/type of PTSD (type 1/type 2). As this may have a very large impact on feasibility, it should be at least reported.

Results: Credibility and satisfaction: you did not include the measures and it is very hard to fathom the informativeness of the values. Measures (CSQ, CEQ) must be referred and you should report cut-off values (e.g. in the Methods) for the different measures.

Discussion/Conclusion: The conclusion TF-CBT is "safe and associated with promising clinical outcomes in a routine care setting" is much too strong, when considering a pre-post design with 22 participants. The conclusion must be re-written with a lot more restraint.

VERSION 1 - AUTHOR RESPONSE

REVIEWER 1

Prof. David Richards, University of Exeter

Comments to the Author:

This study is written as a feasibility study, but the authors fall into the common trap of assigning importance to their clinical outcomes, well beyond what they should do; in effect treating the analysis as if it was a fully powered controlled study. The balance should be tilted more towards feasibility, including progression criteria. I have provided detailed suggestions on this major point and others below.

Response:
Thank you for this important comment. We agree that feasibility studies should primarily emphasize feasibility and progression criteria rather than draw conclusions about clinical effectiveness. In response, we have revised the manuscript to strengthen the focus on feasibility outcomes and to moderate the interpretation of clinical outcomes throughout the paper. These revisions are addressed in the specific comments below.

Comment:
p5, L39. Uncontrolled within group effect sizes cannot be used to "inform...power calculations" for an RCT. They can only be used to acknowledge whether or not an intervention can be considered as a candidate for future trials. Undoubtedly they will significantly over-estimate effect sizes and the variance around the mean will be too large for any meaningful inferences from such a small population. Please rewrite more conservatively.

Response:
Thank you for this comment. We agree that the previous wording could be interpreted as

implying that the uncontrolled within-group estimates could inform power calculations for a future trial. As suggested, we have revised the wording to reflect a more cautious interpretation of these exploratory analyses. The revised text now emphasises that the analyses were intended to provide an initial indication of potential clinical change and to assess whether the intervention appears suitable for further evaluation in a future randomised controlled trial (lines 120–122).

Comment:
P5, L55. Unclear what definition on "1 month" means. Please clarify here that you mean 1 month post the end of treatment. Also, this text would be better moved to the section on measures. It does not belong here.

Response:
Because the timing of the follow-up assessment is specified in the Methods section, where the 1-month follow-up is described as the prespecified primary endpoint, we chose to remove the section referred to above to improve clarity and avoid repetition.

Comment:
P6, L6: ethics should be moved to the separate heading on P10

Response:
The ethics statement has been moved to the separate Ethics section (lines 466–470).

Comment:
P8, L37: intervention description. It is not very clear how long the intervention lasts. Please provide more clarity here.

Response:
We agree that the duration of the intervention should be clearly specified. We have therefore added that the programme was delivered over 12 weeks in the first sentence of the Intervention section (line 235).

Comment:
P8, L49 "vivo" should be "in-vivo"

Response:
We have changed this accordingly (line 242).

Comment:
P9, L23. It is not true to say that blinding was not feasible because of the non-randomised design. Randomisation is irrelevant. It is more correct to state it is an uncontrolled design.

Response:
We agree that the previous wording was imprecise. The text has been revised to clarify that outcome assessors were not blinded due to the uncontrolled study design rather than the absence of randomisation (lines 262-263).

Comment:
P9, L26. The section on PPI is inadequate. Far more detail is required to quantify and qualify PPI activity.

Response:
We have revised this section to provide a more detailed account of how stakeholders were involved throughout the development of the intervention and study (lines 264–286). We now clarify that the intervention originated from clinical needs identified by practitioners, that clinicians with extensive experience working with trauma-exposed youth developed the treatment model, and that young people with and without lived experience of PTSD reviewed materials and provided feedback on content, language, and usability. We also describe consultation with national clinical experts in trauma-focused treatment for children and young people.

We acknowledge that PPI in this feasibility study was more limited than we would ideally have aimed for, partly due to the absence of dedicated funding for structured PPI activities at this stage. We now clarify this in the manuscript and note that a more extensive PPI process is planned for the subsequent randomized controlled trial.

Comment:
P10. Statistical methods. The biggest flaw in the paper is the lack of any mention of progression criteria, nor their analysis, ordinarily recommended using a quantified traffic light system. Why was this approach, recommended by most feasibility guidance, not undertaken? Mellor et al's paper is instructive here: 2023 Apr 15;9(1):59. doi: 10.1186/s40814-023-01291-5. See also my comments on the analysis of clinical outcomes below.

Response:
In this study we did not have explicit progression criteria, which we acknowledge as a limitation (lines 425-429).

Comment:
P11, L46 onwards. The attention given to "clinical outcomes" here is overdone. A tiny uncontrolled feasibility study cannot be analysed to this depth against clinical outcomes. Effect sizes are irrelevant other than to show that the intervention COULD be a candidate for further investigation using better designs. My advice would be to reduce this section of the results very significantly and merely use the post/follow up data to provide an indication of the potential of the treatment. Of more use to the reader and future trial funder would be to know how the end point results compare against a clinical and non clinical population. How many of these participants are outside clinically significant parameters at treatment completion, for example? How many still meet diagnostic criteria? The authors are over-analysing and over-promising from their clinical outcomes data.

Response:
We agree that the emphasis on clinical outcomes in the original manuscript may have been disproportionate given the small uncontrolled feasibility design. In response, we have revised this section of the Results to substantially reduce the level of statistical interpretation and to

frame the findings more clearly as exploratory indications of potential clinical change rather than evidence of treatment effectiveness. Specifically, we have shortened the description of the clinical outcomes and avoided detailed interpretation of individual statistical results in the text, referring readers to the tables for full information. We have also clarified that reductions in participant-reported PTSD symptoms were smaller than those observed in clinician-rated outcomes.

Following the reviewer's suggestion, we now additionally report post-treatment diagnostic status based on the CAPS-CA-5 assessment, including the proportion of participants who no longer met diagnostic criteria for PTSD at the 1-month follow-up.

*Finally, we have revised the wording regarding follow-up to avoid overstating the level of retention and now report the follow-up rate descriptively (17 of 22 participants with available outcome data). These revisions have been implemented in the Results section (lines 349–366)***.**

Comment:
I also note that the participant assessed (CATS - 2 Child) results are far less impressive than clinician rated measures and only 17/22 participants provided post/follow up measures. PROMS of this nature are the gold standard. I find it hard to believe the difference between baseline and post/FU was as large as the authors state in the results from the table. Once again, what do the post/FU measures mean in terms of post-treatment psychopathology compared to a standard population? Why is level of follow up considered to be so wonderful? It is actually a fairly significant loss of end point data.

Response:
Thank you for this comment. As noted in our response to the previous comment, we have revised the Results section to substantially reduce the emphasis on statistical interpretation and to present the findings as exploratory indications of potential clinical change. The revised text (lines 349–366) now provides a more descriptive summary of the outcomes and avoids overinterpretation of the results.

We also clarify that reductions in participant-reported PTSD symptoms were smaller than those observed in clinician-rated outcomes and report follow-up descriptively (17 of 22 participants with available outcome data). In addition, we now report the proportion of participants who no longer met diagnostic criteria for PTSD at the 1-month follow-up.

We also acknowledge that the 23% loss to follow-up represents a limitation of the current feasibility study. In the Discussion, we now note this and outline potential strategies to improve retention in a future RCT. Primarily, this involves asking the adolescent to complete this and allowing time for it before conducting the CAPS-CA-5 with the adolescent, rather than only reminding them afterwards. These measures are intended to support higher retention while remaining feasible within routine care settings.

Comment:
P12: discussion. It is very difficult to justify the strong statements on outcomes used by the authors without any progression criteria being specified. Against what criteria is the

intervention "feasible and well accepted"?` The same critique applies to the first part of the subsequent para (L50 onwards). These are strong statements pulled out of the air with no a priori criteria specified against which to make these assertions. This is a tiny study, uncontrolled, from a volunteer population with less than 15% of people initially expressing interest taken into the study. The authors overstate their case very much.

Response:
In this study we did not have explicit progression criteria, which we acknowledge as a limitation (lines 425-429). We have also adjusted the language according to the reviewer's comment (lines 372-375 and 432-435)

Comment:
In Table 2 the description of initiating traumatic events includes highly specific ones and general descriptions such as bullying. Surely there is a lot of overlap here. Of course, the initiating event is less important (apart from as a focus of treatment) than the clinical presentation, but nonetheless I will not be the only reader who found this list arbitrary.

Response:
Thank you for this observation. The categories presented in Table 2 reflect how the initiating traumatic events were described in the clinical assessments (CAPS-CA-5) rather than a predefined research classification system. Consequently, the level of specificity varies across categories and some conceptual overlap is possible (e.g., between bullying and other forms of interpersonal violence). The purpose of the table was primarily descriptive, to give an overview of the types of events reported by participants, rather than to present mutually exclusive trauma categories. We have clarified this in the manuscript to make this more explicit for the reader (lines 220-223).

REVIEWER 2

Mr. Florian Hammerle, University Medical Center of the Johannes Gutenberg University Mainz

Comments to the Author:

Thank you for the opportunity to review the manuscript " Therapist-guided internet delivered trauma-focused CBT for adolescents with PTSD: A feasibility trial". The manuscript is of interest and I have great respect for the commitment that went into the study.

Nevertheless, I have a lot of major concerns and concerns about the manuscript:

Response:
We thank the reviewer for their careful and constructive assessment of our manuscript and for highlighting important concerns. These comments have helped us strengthen and clarify the manuscript, and we address each point in detail below.

Comment:
A main issue is that some portions of the manuscript are misleading. If I would only read the

title/abstract, I would expect that the therapy was delivered synchron, but the main the treatment was asynchron (and in large portions not asynchrony but only processed by patients/caregivers/legal guardians themselves) and via a messaging system (neither voice by call nor video). As this seems very important, it should be pointed out throughout the manuscript (and in title/abstract) and the manuscript should be re-written according to this.

Response:
We agree that the original wording may have given the impression that the treatment was delivered synchronously. In the present intervention, therapist support was primarily delivered asynchronously through secure messaging on the treatment platform, with therapists providing written feedback and guidance on completed exercises. Synchronous contact (e.g., telephone or video) was available if needed but was not a core component of the intervention.

To clarify this, we have revised the manuscript to more explicitly describe the asynchronous, messaging-based format of the intervention. Specifically, the Abstract has been revised (line 48) and the Intervention section has been clarified (lines 246) to indicate that therapist guidance occurred primarily through asynchronous messaging on the platform.

Comment:
E.g. in the introduction (page 5, lines 21-30) treatment efficacy of internet delivered CBT is referred to a meta-analysis which was based on synchron treatment, but I could point a lot of other parts of the manuscript, where these inconsistencies are reported.

Response:
Thank you for highlighting this inconsistency. We agree that the introduction could more clearly distinguish between synchronous and asynchronous formats of internet-delivered CBT. We have therefore revised the wording in this section (lines 109-110) to ensure that the description of previous research aligns more accurately with the delivery format used in the present study.

Comment:
This lead to a second major issue: some portions of the manuscript are not carefully elaborated and written. E.g. the before mentioned argument, that internet-based therapy may be effective is based on three references:

11. Vigerland S, Lenhard F, Bonnert M, et al. Internet-delivered cognitive behavior therapy for children and adolescents: A systematic review and meta-analysis. Clinical Psychology Review. 2016/12/01/ 2016;50:1-10. doi:https://doi.org/10.1016/j.cpr.2016.09.005

12. Pynoos RS, Weathers, F. W., Steinberg, A. M., Marx, B. P., Layne, C. M., Kaloupek, D. G., Schnurr, P. P., Keane, T. M., Blake, D. D., Newman, E., Nader, K. O., & Kriegler, J. A. . Clinician-Administered PTSD Scale for DSM-5 - Child/Adolescent Version. . 2015.

13. Sheehan DV, Sheehan KH, Shytle RD, et al. Reliability and validity of the Mini International Neuropsychiatric Interview for Children and Adolescents (MINI-KID). J Clin Psychiatry. Mar 2010;71(3):313-26. doi:10.4088/JCP.09m05305whi

But only the first reference refers to a meta analysis and citation 12 and 13 refer to instruments.

Response:
We believe there may have been a misunderstanding regarding the cited references. References 11–13 in the submitted manuscript do not correspond to the references described by the reviewer, but instead refer to the studies listed below, all of which concern internet-delivered treatments for PTSD.

If there has been a technical issue affecting the reference list, we would of course be happy to clarify or provide additional information.

11. *Bisson JI, Ariti C, Cullen K, et al. Guided, internet based, cognitive behavioural therapy for post-traumatic stress disorder: pragmatic, multicentre, randomised controlled non-inferiority trial (RAPID). BMJ 2022;377:e069405. doi:10.1136/bmj-2021-069405*
12. *Bragesjö M, Arnberg FK, Olofsdotter Lauri K, et al. Condensed internet-delivered prolonged exposure provided soon after trauma: a randomised trial. Psychol Med 2023;53(5):1989–98. doi:10.1017/s0033291721003706*
13. *Simon N, McGillivray L, Roberts NP, et al. Acceptability of internet-based cognitive behavioural therapy (i-CBT) for post-traumatic stress disorder (PTSD): a systematic review. Eur J Psychotraumatol 2019;10(1):1646092. doi:10.1080/20008198.2019.1646092*

Comment:
This is also true for terminology. In the abstract, participants are described as „adolescents" but in the strengths and limitations the first strength is stated as "TF-CBT trial for young people". In the Methods' section, under study design, you refer to "Save the Children Sweden" (which I understand is a proper noun) but go on describing clients as "children" (which is another key term than adolescents, young people etc.) and under "Participants" you also use the term "both children and parents" [whereas I am not shure, if all were in fact parents or caregivers/legal guardians]. This is a major inconsistency throughout the manuscript (e.g. also in acceptability and satisfaction "children", safety "adolescents" and elsewhere). I would recommend to use a stringent wording (e.g. stick to the term "adolescents" throughout the manuscript.

Response:
Thank you for highlighting this important point. To address the inconsistency, we have carefully reviewed the manuscript and now consistently use adolescents when referring to study participants and caregivers when referring to participating adults. These terms are applied throughout the manuscript to ensure clarity and uniform terminology.

Comment:
As another, but not exclusively, example of inconsistencies, in the "Study, design and

setting", aspects of the sample/measures (Independent evaluators administered the CAPS-CA-5 at baseline and at 1-month, the prespecified primary endpoint.) are described, which should be moved to other sections of the manuscript (as they are neither study design nor setting).

Response:
Thank you for highlighting this. We have removed this accordingly since this information was already provided at in other sections.

Comment:
This is also true for AE and especially SAE. You report AE and SAE in the results but do not inform in the method how they were measured and especially the categorization as SAE is not included in the method. Skipping your definition and the measurement of SAE (I expect suicide attempt/death, hospital admission >2 days but probably not birth anomaly as I should not be expected to be triggered by psychotherapy etc.) is not acceptable in a manuscript (especially when dealing with participants with PTSD and higher risk for SAE) and must be augmented.

Response:
Thank you for this helpful comment. We have added explicit definitions of adverse events (AEs) and serious adverse events (SAEs) to the Methods section and clarified how these were monitored throughout the study (lines 179–184). This addition ensures that the categorisation and assessment of AEs and SAEs are now fully described in accordance with standard reporting practice.

Comment:
I don't really find the strength that the "intervention was developed in close collaboration with clinicians" really important (whereas the participation of young people [please refer to my before mentioned issue of key terms] could be) because most interventions are developed in close collaboration to clinicians. I would rather recommend use the independency of treatment deliverance and assessors or other aspects.

Response:
We thank the reviewer for this comment. We have revised the Strengths and Limitations section and removed the statement regarding the intervention being developed in collaboration with clinicians.

Comment:
The first paragraph of the introduction is poorly designed. The first sentence is misleading as the two cited papers [which are somewhat old] don't report point prevalence rates, but lifetime prevalence. Therefore, violence and traumatic events are not only estimated in the two papers within adolescence but in adolescents, who may have experienced trauma as children. You switch between these prevalence rates of trauma in adolescents, refer to prevalence of PTSD in children and use a citation, which refers to mental illness in refugees to point out an increasing number of community shootings. If I search Wikipedia for community shootings in Sweden, I get this list:

10 January 2004 Knutby, Uppland

1 1 Knutby murder: Alexandra Fossmo was killed. Her employer, 30-year-old IT entrepreneur Daniel Linde, was shot in the head and chest, but survived.[8][9]

18 March 2015 Gothenburg

2 8+ 2015 Gothenburg pub shooting: A gang-related shooting occurred in which a 25-year-old said to be a leading figure in a local gang and a 20-year-old bystander were killed.

2 August 2020 Norsborg, Botkyrka

1 0 Murder of Adriana Naghei Ostrowska: A 12-year-old girl named Adriana was shot dead in an attempted mass shooting.[10]

10 April 2024 Skärholmen, Stockholm

1 0 Murder of Mikael Janicki: A 39-year-old man named Mikael Janicki was shot dead in a pedestrian tunnel in front of his 12-year-old son after confronting a group of youths in Skärholmen, Stockholm.[11]

4 February 2025 Örebro, Närke

11[b]

6 Örebro school shooting: 35-year-old Rickard Andersson shot and killed 10 students and injured 12 others before committing suicide.[12]

29 April 2025 Uppsala, Uppland

3 0 2025 Uppsala shooting: Three people were killed on the eve of the Walpurgis festival.[13] A 16-year-old male suspect was taken into custody.

Although I understand, that there is an increase in community shootings, the absolute prevalence of community shootings is very low and I am in doubt if these isolated shootings (as bad as they are and I am sorry for every dead/harmed person and the relatives/friends) are the main cause of PTSD or a large increase in numbers of PTSD in Sweden.

Even more, it does not seem necessary to point out, that there might be an increase in PTSD prevalence rates as the prevalence rates are already high enough and Sweden is a country with a relatively small population and large distances between many people. Together with the skepticism of adolescents with PTSD in regard to therapy (e.g. due to shame and guilt) this may be enough to argue for a TF-CBT delivered by internet.

All in all, the complete first paragraph should be redesigned to follow a better red thread.

Response:
Thank you for this detailed and helpful comment. We agree that the first paragraph lacked clarity regarding the epidemiological framing and could be improved in terms of structure. We have therefore revised the paragraph to clarify that the cited studies refer to cumulative exposure to traumatic events by late adolescence rather than point prevalence during adolescence.

We have also standardized the terminology used to describe the population and improved the logical flow of the paragraph. The revised version now first introduces the prevalence of exposure to potentially traumatic events, followed by the prevalence of PTSD among trauma-exposed youth, and subsequently the clinical consequences of PTSD.

Finally, we have removed the sentence referring to community shootings in Sweden, as we agree that this example was not essential for the rationale of the study. The paragraph has therefore been substantially rewritten to provide a clearer and more coherent introduction to the topic (line 87-90).

Comment:
Participants: how did you evaluate the suicide risk? Did you use any kind of interview (such as the C-SSRS)? If not, this should be described and mentioned as limitation.

Response:
Suicide risk was assessed as part of the baseline diagnostic assessment using the MINI-KID (Procedure section). To improve clarity, we have revised the text to more explicitly indicate that suicide risk assessed during the MINI-KID interview was considered when determining eligibility (line 219 and 225 in the revised manuscript).

Comment:
There is no record of trauma/type of PTSD (type 1/type 2). As this may have a very large impact on feasibility, it should be at least reported.

Response:
Information on trauma type was collected as part of the demographic and clinical information obtained from adolescents at screening (see Procedure section) and is reported in Table 2. PTSD diagnoses were established using the CAPS-CA-5 according to DSM-5 criteria. The classification of PTSD into "type I" and "type II" was not applied, as this distinction is not part of the DSM-5 diagnostic framework used in the present study.

Comment:
Results: Credibility and satisfaction: you did not include the measures and it is very hard to fathom the informativeness of the values. Measures (CSQ, CEQ) must be referred and you should report cut-off values (e.g. in the Methods) for the different measures.

Response:
The measures used to assess credibility/expectancy and treatment satisfaction (Credibility/Expectancy Questionnaire, CEQ, and Client Satisfaction Questionnaire, CSQ-8) are described in the Methods section, including the number of items and score ranges. These measures are typically analysed as continuous scales and established cut-off values for interpretation are generally not used. To further improve clarity, we have revised the Results section to explicitly indicate the instruments used when reporting the results (lines 327–328 in the revised manuscript).

Comment:
The conclusion TF-CBT is "safe and associated with promising clinical outcomes in a routine

care setting" is much too strong, when considering a pre-post design with 22 participants. The conclusion must be re-written with a lot more restraint.

Response:
We agree that conclusions from a small single-group feasibility study should be interpreted with caution. We have therefore revised the Conclusions section to use more cautious language and to emphasise that the observed symptom improvements should be interpreted as preliminary given the small sample size and uncontrolled design (line 431-435).

VERSION 2 - REVIEW

**Reviewer**	**1**
**Name**	**Richards, David**
**Affiliation**	**University of Exeter, Institute for Health Research**
**Date**	**07-Apr-2026**
**COI**	

I believe that you have carefully and appropriately addressed my review comments and I have no further suggestions.
